# Psychomotor Agitation Non-responsive to Treatment: A Case Report of Phenibut Withdrawal Syndrome

**DOI:** 10.3389/fpsyt.2021.688147

**Published:** 2021-06-28

**Authors:** Cecilia Maria Esposito, Gian Mario Mandolini, Giuseppe Delvecchio, Alessio Fiorentini, Paolo Brambilla

**Affiliations:** ^1^Department of Neurosciences and Mental Health, Fondazione Istituto di Ricovero e Cura a Carattere Scientifico Ca' Granda, Ospedale Maggiore Policlinico, Milan, Italy; ^2^Department of Pathophysiology and Transplantation, University of Milan, Milan, Italy

**Keywords:** withdrawal, psychiatric aspects, psychopharmacology, phenibut, psychomotor agitation

## Abstract

**Background and Objectives:** Phenibut (4-amino-3-phenyl-butyric acid), acting as a GABA-B receptor agonist, has a beneficial effect on anxiety. Although its medical use is not approved in western countries, it can be easily obtained worldwide *via* the Internet, so it spread as a substance of abuse. In recent years, some case reports have, therefore, highlighted episodes of acute toxicity or withdrawal, but it is still a largely unknown phenomenon.

**Methods:** In this case report, a 50-year-old woman was admitted to the emergency room with psychomotor agitation, psychotic symptoms, and insomnia, and was non-responsive to treatment. She was hospitalized at the psychiatry ward for 25 days and gave her consent for the publication of the present case report.

**Results:** The suspicion of phenibut withdrawal allowed to establish the appropriate management, leading to the *restitutio ad integrum* of the psychopathological case.

**Conclusions:** In the face of an incoercible psychomotor agitation case, the knowledge of the so-called novel psychoactive substances allows for more appropriate clinical management of intoxication and withdrawal syndromes. This is a scientifically significant report as it provides therapeutic and outcome data concerning a syndrome that is still quite unfamiliar.

## Introduction

Phenibut (4-amino-3-phenyl-butyric acid) is a glutamic acid derivative compound synthesized in Russia in the early 1960s and available nowadays in ex-Soviet countries as a cognitive enhancer, food supplement, adjuvant for anxiety and insomnia, and alcohol withdrawal symptoms (1). This substance seems to primarily act as a γ-aminobutyric acid (GABA) B receptor agonist, consequently closing voltage-dependent calcium channels and inhibiting neurotransmission, similar to other drugs, such as pregabalin, gabapentin, and baclofen (2). Moreover, phenibut seems also to boost both dopaminergic and serotoninergic neurotransmission (3). The pharmacological characteristics of phenibut can be viewed in Table 1. However, even though its medical use is not approved in western and European countries, since it was classified as a novel psychoactive substance (NPS) by the United Nations Office of Drug and Crime (UNODC), phenibut can be easily obtained worldwide *via* the Internet as a dietary supplement in the form of powder, pills, or crystals with an increasing risk of potential misuse (4). In this regard, both acute intoxication and withdrawal syndromes related to phenibut consumption have been reported in literature (5). Specifically, intoxication mainly induces the risk of respiratory failure, paradoxical agitation, seizures, and delirium, while withdrawal is a condition that can last for a significant period and is characterized by psychomotor agitation, psychosis, autonomic instability, seizures, nausea, and vomiting (6, 7). These clinical conditions must be timely recognized and treated in order to avoid serious complications, such as respiratory or acute renal failures due to rhabdomyolisis (5). However, the clinical manifestation characterized by non-specific signs and symptoms together with the lack of a specific protocol for the treatment of both phenibut intoxication and withdrawal symptoms could delay the recognition of these syndromes and their effective management. Therefore, the description of case reports related to phenibut misuse is crucial in order to make clinicians aware of this emerging NPS misuse.

**Table 1:** Phenibut: chemical and pharmacological characteristics.

**Phenibut, Anvifen, Fenibut, Noofen**	
4-Amino-3-phenyl-butyric acid Chemical structure: C_10_H_13_NO_2_	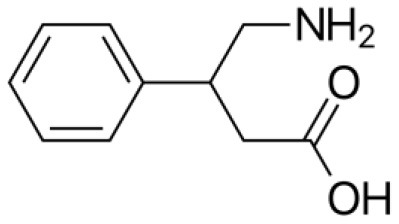
Pharmacological characteristics: -GABA-mimetic, primarily at GABA(B) and, to some extent, at GABA(A) receptors -Stimulator of dopamine receptors and antagonizes beta-phenethylamine, a putative endogenous anxiogenic -Blocker of α2δ subunit-containing voltage-dependent calcium channels	

## Case Report

**Figure 1:**
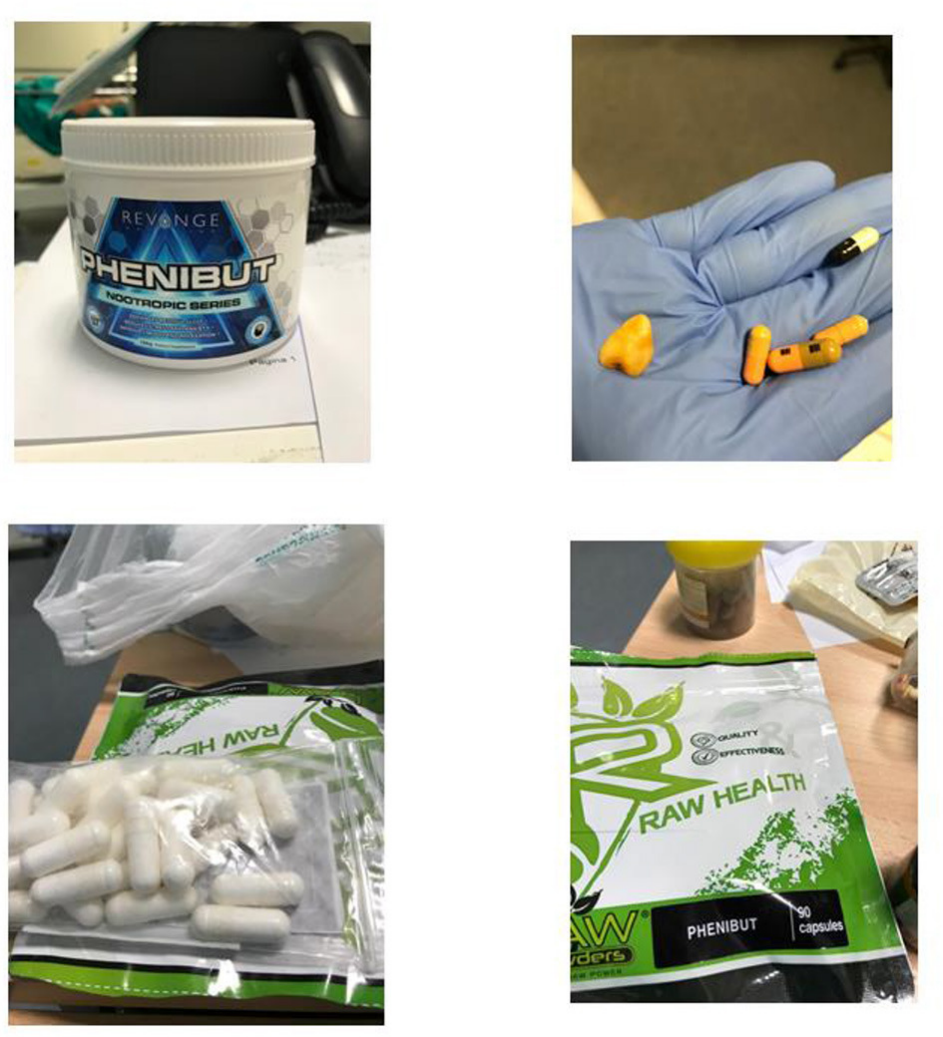
Pictures of phenibut tablets in various commercial formulations (Fenibut, Anvifen, and Noofen) found at patient's home.

A 50-year-old woman with previously unknown psychiatric history was admitted to the emergency department at night in a state of confusion and psychomotor agitation. Her partner declared that during the morning, the patient suddenly developed motor stereotypies, hyperactivity, and fluctuations of both attention and consciousness. Although her partner denied that the patient had used any psychoactive drugs or alcohol previously, he reported an occasional consumption of diazepam oral solution for anxiety. The patient was not taking any drug therapy with medical prescription. Since psychomotor agitation was becoming more severe with the patient's risk of self-injurious conduct, intramuscular medication with delorazepam up to 6 mg was administered without any substantial modification of the symptomatology. Meanwhile, a CT scan without contrast, performed at the emergency room, was negative for acute neurological events, while toxicological screening of urine (research for opioids, cocaine, methamphetamine, cannabinoids, and benzodiazepines) was positive only for benzodiazepines. The patient's blood tests showed no significant alterations except for creatine phosphokinase (CPK), while the electrocardiogram detected no significant alterations except for a tachycardia (120 beats for a minute). After 2 h of intramuscular therapy with benzodiazepines, haloperidol, and promazine, the patient had no clinical improvement showing abnormal motor behaviors, disorganized thinking, echolalia, visual hallucinations, and total insomnia. Her partner was able to recover a series of tablets at home, of which phenibut, in its various commercial formulations (Fenibut, Anvifen, and Noofen) was the main ingredient (Figure 1). Upon contacting the Poison Control Center, the clinical symptomatology presented by the patient was suspected to be related to phenibut withdrawal since the patient had started consuming phenibut in the previous months. It was subsequently possible to reconstruct that the interval between the last dose of phenibut and the onset of symptoms was about 3 days. The patient was, therefore, hospitalized in the psychiatric ward. Meanwhile, intravenous diazepam up to 30 mg and intramuscular haloperidol up to 5-mg therapy was administered. Following the recommendations for phenibut withdrawal syndrome from previous case reports (5), a baclofen medication of up to 20 mg/day was started. This is because previous literature reported baclofen as a GABA-B agonist, which allows an alternative binding of GABA-B receptors and, therefore, an improvement on withdrawal (8). A time course regarding the drug treatment and the dosages used is shown in Table 2.

**Table 2:** Timeline regarding drug treatment and dosages.

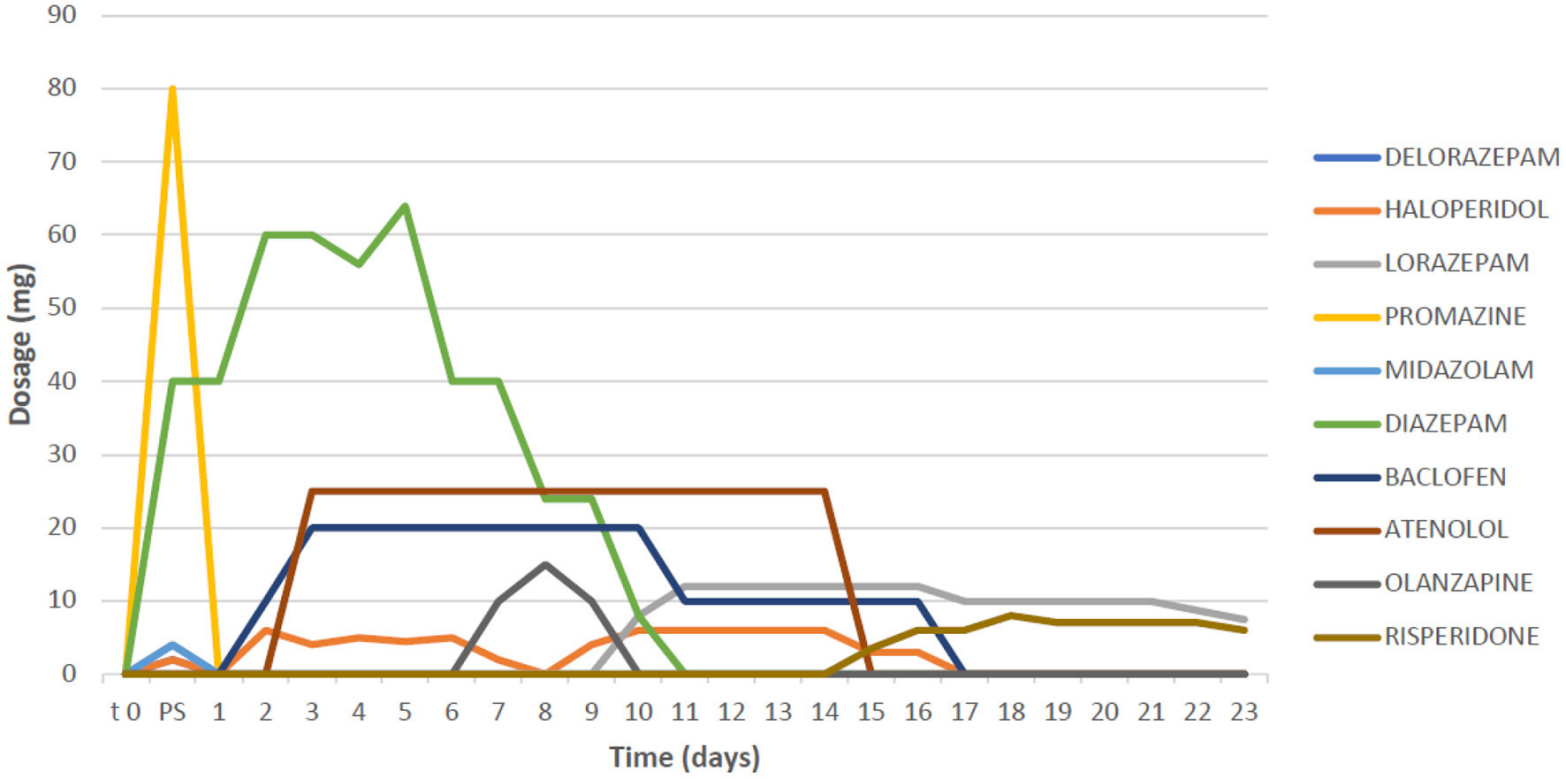

Despite the therapy, the patient still spent two completely sleepless nights, experiencing visual hallucinatory disturbances, disorganized behavior, and thinking, with no clearly structured delusions. Psychometric rating scales were performed, with evidence of significant alteration of the mental state [Brief Psychiatric Rating Scale (BPRS) = 75, Hamilton Rating Scale for Depression (HAM-D) = 24, Mania Rating Scale (MRS) = 22, Positive and Negative Syndrome Scale (PANSS) = 102, and Global Assessment of Functioning (GAF) = 25]. Afterward, her mental status began to change from agitation, self-directed aggressiveness, and persecutory delusions to episodes of catatonia, during which she did not react to stimuli and appeared hostile and opposed to any therapeutic contact. Electroencephalogram (EEG) and magnetic resonance imaging (MRI) were performed, with the former showing rapid rhythms compatible with benzodiazepine therapy, and the latter exhibiting rare punctiform hyperintense signal alterations in T2-FLAIR affecting the bihemispheric subcortical white matter of non-specific gliotic significance. In the context of catatonia, the patient developed bladder globe and urinary tract infection with the consequent need for antibiotic treatment (ceftriaxone 2 g for 6 days). She never showed signs of kidney damage, and there was a progressive decrease in CPK (from 1,504 to 195 U/L). Instead, a picture of autonomic instability emerged, characterized by pressure peaks and tachycardia; therefore, atenolol treatment up to 10 mg/day was started, and this had positive effects on symptoms.

Meanwhile, it was possible to view previous health records, and the patient's medical history was reconstructed. She did not suffer from any major medical diseases, but she had been previously treated by private psychiatrists at the age of 36, for depressive episodes in the context of bipolar disorder with psychotic features. She had not been working for 20 years, and she had been living with her partner, living a mainly solitary life with few social interactions. Complete intercritical resolution of the depressive episodes was reported, with a return to the previous functioning. However, for cultural reasons, the patient continued to have magical thoughts. Amitriptyline and benzodiazepines were the last pharmacological therapy administered, prescribed 3 years before the current episode by a private psychiatrist and consumed by the patient without any medical supervision, which, due to her history of poor pharmacological compliance and her tendency to prefer natural remedies, may have not been taken correctly. She had no history of substance abuse, although a trend of excessive consumption of benzodiazepines was also reconstructed for anxiolytic and hypno-inductive purposes.

In light of the catatonic state, the therapy was changed from diazepam to intravenous lorazepam up to 12 mg/day (9). Furthermore, since occasional lengthening of the QT interval was detected through ECG, haloperidol was replaced first with olanzapine, then with risperidone up to 6 mg in order to facilitate the management of psychomotor agitation with a daily QT monitoring. Gradually, the patient progressively showed a reduction in both disorganized thinking and agitation. In addition, psychotic symptoms, such as persecutory delusions and both visual and auditory hallucinations, slowly diminished until finally ending after 4 weeks. Atenolol therapy was stopped after 15 days, and the patient did not experience any further symptoms of autonomic instability.

After resolving the psychotic symptomatology, the patient showed positive recovery in regard to delusional thinking and hallucinatory phenomena, but she also experienced a few days of moderate expansive mood, which resolved after a few days. The patient revealed that she had been consuming phenibut in high dosage (up to 5 g/day) in the previous months in order to treat anxiety and insomnia that began during the COVID-19 pandemic quarantine. Therefore, the diagnosis of phenibut withdrawal was confirmed. Finally, psychometric rating scales were performed at the end of the hospitalization showing the following results: BPRS = 25, HAM-D = 5, MRS = 2, PANSS = 37, GAF = 80. We concluded on a diagnosis of withdrawal psychosis and mixed psychotic episode in the context of bipolar disorder. The patient was, therefore, discharged after 25 days of hospitalization, with a diagnosis of withdrawal psychosis and mixed psychotic episode in bipolar disorder, and with the following treatment: risperidone 6 mg and lorazepam 10 mg/day.

Although it was impossible to have a detailed view of her perspective during the entire hospitalization, at the time of discharge, the patient expressed feelings of relief and amazement concerning her well-being. She also said that she had lived “a nightmare” and that she not only had fear but also, in some moments, the certainty that it would never end. The patient gave her informed consent for the publication of the present case report.

## Conclusions

This case report aims to underline the disruptive action that NPS can have in the psyche of a subject, especially due to intoxication and abstinence. In this case, surely the duration of the episode is not to be attributed only to the severity of the condition of abuse but also to the presence of the patient's previous psychiatric disorder. In fact, the previous diagnosis of bipolar disorder may have affected both the emotional instability, which pushed the patient toward the abuse of phenibut, and the severity of the consequent psychopathological picture (10). Moreover, in this patient, it seems that the abuse was not determined by a sensation-seeking modality but by the inability to manage feelings of emptiness and fear due to the COVID-19 pandemic emergency that recently occurred in northern Italy (11, 12). The observation of the exotoxic origin of the very serious episode of psychosis described in this case report creates an interesting field of investigation with respect to the so-called synthetic psychosis. This has led to a great diffusion in recent years and, thus, has made it important for knowledge to be acquired on the phenomenon to enable its differentiation from non-exotoxic psychiatric disorders (13, 14).

The emerging worldwide misuse of phenibut (an NPS inaccurately marketed as a dietary supplement) requires major attention from clinicians in order to recognize both its intoxication and abstinence syndromes, which are two clinical conditions that can be characterized by initial slow response to multiple treatments and several serious life complications. Finally, given its various pharmacological actions with potential for tolerance and withdrawal, phenibut should be considered a substance requiring close medical supervision, and its prescription should be regulated by competent medical authorities.

## Data Availability Statement

The original contributions presented in the study are included in the article/supplementary material, further inquiries can be directed to the corresponding author.

## Ethics Statement

Written informed consent was obtained from the individual(s) for the publication of any potentially identifiable images or data included in this article.

## Author Contributions

AF and PB conceived the presented idea. CE and GM wrote the manuscript in consultation with GD. PB supervised the project. All authors contributed to the article and approved the submitted version.

## Conflict of Interest

The authors declare that the research was conducted in the absence of any commercial or financial relationships that could be construed as a potential conflict of interest.
